# Paradoxical Facilitation of Working Memory after Basolateral Amygdala Damage

**DOI:** 10.1371/journal.pone.0038116

**Published:** 2012-06-08

**Authors:** Barak Morgan, David Terburg, Helena B. Thornton, Dan J. Stein, Jack van Honk

**Affiliations:** 1 MRC Medical Imaging Research Unit, Department of Human Biology, University of Cape Town, Cape Town, South Africa; 2 Department of Psychology, Utrecht University, Utrecht, The Netherlands; 3 Department of Psychiatry and Mental Health, University of Cape Town, Cape Town, South Africa; Charité University Medicine Berlin, Germany

## Abstract

Working memory is a vital cognitive capacity without which meaningful thinking and logical reasoning would be impossible. Working memory is integrally dependent upon prefrontal cortex and it has been suggested that voluntary control of working memory, enabling sustained emotion inhibition, was the crucial step in the evolution of modern humans. Consistent with this, recent fMRI studies suggest that working memory performance depends upon the capacity of prefrontal cortex to suppress bottom-up amygdala signals during emotional arousal. However fMRI is not well-suited to definitively resolve questions of causality. Moreover, the amygdala is neither structurally or functionally homogenous and fMRI studies do not resolve which amygdala sub-regions interfere with working memory. Lesion studies on the other hand can contribute unique causal evidence on aspects of brain-behaviour phenomena fMRI cannot “see”. To address these questions we investigated working memory performance in three adult female subjects with bilateral basolateral amygdala calcification consequent to Urbach-Wiethe Disease and ten healthy controls. Amygdala lesion extent and functionality was determined by structural and functional MRI methods. Working memory performance was assessed using the Wechsler Adult Intelligence Scale-III digit span forward task. State and trait anxiety measures to control for possible emotional differences between patient and control groups were administered. Structural MRI showed bilateral selective basolateral amygdala damage in the three Urbach-Wiethe Disease subjects and fMRI confirmed intact functionality in the remaining amygdala sub-regions. The three Urbach-Wiethe Disease subjects showed significant working memory facilitation relative to controls. Control measures showed no group anxiety differences. Results are provisionally interpreted in terms of a ‘cooperation through competition’ networks model that may account for the observed paradoxical functional facilitation effect.

## Introduction

Working memory is intimately related to attention, so much so that they are sometimes fused into the single concept of “working attention” [1] and it has recently been proposed that working memory is in fact nothing more than “flexibly deployable attention” [2]. At the neural level this means that information held “in” working memory is not stored anywhere other than in sensory or other representational systems (e.g. motor-planning, motor-control, speech production and comprehension) that generate it in the first place. In this view control of WM is no different from executive control in general [2], [3]. fMRI studies indicate that executive functions are subserved by a distributed ‘central executive network’ wherein dorsolateral prefrontal cortex (DLPFC) and parietal cortex play pivotal roles [3]–[5]. The PFC thus remains a major albeit non-mnemonic locus of working memory operations [2], [3]. Moreover within the PFC, neurons do not appear to make fixed localised contributions to executive function, instead adapting their activity according to current needs [3].

Given the vital survival value of detecting stimuli signalling events such as threats, mates, food etc., the evolution of executive attention arguably entailed new mechanisms for preventing such critical but ultimately distracting information from interfering with voluntary PFC operations. For this reason voluntary control over competing stimuli is considered an essential element of executive control [2]. Whereas previous models of WM interpret PFC neural activity during the delay-period of a WM task as information storage activity, more recent models interpret it as executive control activity serving a variety of functions, none of which are specific to WM and all of which encompass mechanisms that actively sustain selective attention, particularly in the face of competing internal or external interference [2]. Postle refers to this delay-period activity as follows: *“The variously named “guided activation” or “adaptive coding” theories emphasize the role of PFC in biasing stimulus-response circuits such as that over-learned, prepotent associations can be overcome in favour of novel, or otherwise less salient behaviours, thereby enabling flexible behavioural response to unfamiliar or atypical situations”* [2]. Active mechanisms of selective attention can take two forms. On the one hand there is evidence suggesting that PFC activity reflects a “distraction-detection mechanism” that selectively inhibits processing of non-salient information (i.e. non-salient from the perspective of voluntary working attention) in posterior cortex (e.g. sensory cortex) [6]. On the other hand, Sakai et al. [7] found evidence for a PFC-controlled “active maintenance” process that strengthens mnemonic sensory information in posterior cortex during distractions.

From the perspective of working attention, salient information is information most pertinent to current wilful executive goals. The brain however has another ‘salience detector’, a form of effortless, involuntary attention constantly scanning the environment for signs of danger or reward. Numerous neocortical regions are involved in non-executive salience detection [4], [5], [8] but an ancient subcortical structure, the amygdala, has more than any other part of the brain been implicated in automatically orientating attention to the most relevant stimuli [9]–[11]. Similar to how PFC delay-period activity is thought to bias attention towards representations salient to working attention, diverse evidence shows that the amygdala automatically (i.e. always) biases attention towards representations salient to survival by modulating cortical activity at both posterior sensory and executive PFC levels [12], [13].

During WM operations, stimuli considered most salient by the amygdala are frequently likely to be precisely those stimuli considered non-salient by PFC delay-period activity responsible for distractor-detection and sustained selective attention. Yet very little is known about the amygdala in relation to working memory [14]. Available studies show that both increased and decreased amygdala activation is associated with enhanced WM performance depending on task conditions. On the one hand, amygdala fMRI activation correlates positively with performance speed (without effecting accuracy) but only where there is significant task difficulty (i.e. in 3-back versus 1-back WM task). This correlation was found to be independent of emotional, mood or personality factors [14]. It is however unclear whether these findings are specific to WM or merely reflect relations between amygdala activation, speeded response times and cognitive load. Other studies have found the opposite - amygdala deactivation correlates with improved cognitive performance in the context of increased cognitive load [14]–[16].

Only two studies appear to have investigated amygdala activity during WM in the presence of interference. Yun et al. [15] administered 0-, 1-, 2- and 4-back WM tasks while measuring brain fMRI activation (the 4-back task was designed to induce negative affect in participants in response to high error rates). Results showed progressively less amygdala and VLPFC activation with increasing WM load while DLPFC showed the opposite pattern. Notably, while fMRI activity in PFC regions changed monotonically, in the amygdala it showed a pronounced step function: The decrease between 0-back and 1-back being about 20 times smaller than the decrease between 1-back and 2-back. There was no change in amygdala fMRI activation between 2-back and 4-back. This pattern suggests a relatively high level of baseline amygdala activity under conditions of minimal load and no induced affect, i.e. during the 0-back and 1-back conditions. This baseline activity was however markedly suppressed during the more challenging 2-back condition. Although the 4-back condition is much harder than the 2-back, no further suppression of amygdala activity was observed. However, across individuals it was found that failure to suppress amygdala activation at higher WM loads results in poorer performance.

Most notably, this study also found that increased negative coupling between DLPFC and amygdala during a difficult 4-back WM task predicted poorer performance recovery on an easier 2-back task that immediately followed the 4-back task. According to the authors these results indicate that failure to suppress amygdala activation at high WM loads (i.e. 4-back task associated with negative affect) results in strong amygdala-DLPFC coupling indicative of bottom-up emotional interference that persists for some time. Affective factors therefore do seem to bear upon WM performance.

Another recent fMRI study also found that the strength of coupling between blood flow increases in DLPFC and blood flow decreases in the amygdala correlates with better working memory performance. Anticevic et al. [16] investigated the effects of external interference on WM performance and fMRI activity. This study looked at negative, neutral and task-related distractors and found significantly higher levels of amygdala activation was associated with poorer WM performance for all distractor types. At rest there was also negative coupling between amygdala and dorsal executive PFC regions. Notably, negative coupling was significantly greater during WM than at rest for all PFC regions and was again significantly greater during WM with negative distractors. These results reinforce the idea that negative affect interferes with WM performance and while they are also consistent with the idea of PFC activity down-regulating amygdala activity, decreased dorsal (anterior-dorsal and dorsolateral) PFC activity was in fact found to be associated with increased WM performance, specifically for negative distractors. However, the opposite was true for VLPFC where greatest signal increases were associated with better performance in the case of negative distractors.

These studies provide broad support for the idea that amygdala activity impairs WM performance, not only during internal [15] or external [16] negative affect, but also when nothing salient is happening (e.g. neutral distracters, [16]). The fact that a substantial quantum of baseline amygdala activity is suppressed in the transition from a 1-back to a 2-back task administered by Yun et al. [15] suggests that automatic amygdala surveillance mechanisms also consume attentional/processing resources, even at baseline in the absence of salience.

As discussed above PFC delay-period activity is thought to represent executive control including active mechanisms for protecting selective attention from interference. “Distraction-detection”, “active maintenance”, “guided activation” etc. all suggest the PFC must *actively* overcome more automatic modes of cognition in order that working attention (including WM operations) may generate novel, flexible behavioural responses. These considerations together with the observations of Anticevic et al. [16] of weakly negative coupling between amygdala and DLPFC at rest, that increased during WM operations, and increased yet further in the presence of negative distractors all engender the hypothesis that the evolution of executive attention introduced competition for attentional/processing resources between PFC and amygdala, even at baseline in the absence of emotional salience.

All of the above studies of amygdala function in relation to WM speak of the amygdala as a whole, but within the amygdala the vast majority of incoming signals converge on the basolateral complex (BLA) [9], [17], [18]. The BLA functions as a central hub orchestrating the activity of multifarious cortical and subcortical networks to ensure continual detection, evaluation, communication and regulation of salient information [9], [17]–[22]. The BLA lies between and displays cytoarchitectural characteristics inbetween isocortex and subcortex [23] making it well-suited for this role. On one hand it is cortical-like and receives massive unimodal and polymodal cortical sensory inputs [9], [17], [18], [24] as well as higher-order cognitive “knowledge” from the prefrontal cortex (PFC) [8]. Similarly, most of the fibers projecting from amygdala to the cortex stem from the BLA, particularly targeting sensory association cortex, orbitofrontal cortex (OFC) and, in primates, primary sensory cortex [18]. The BLA also modulates cortical arousal or attentional vigilance via cholinergic and other basal forebrain nuclei [17]. Lastly, the BLA shares rich bidirectional connections with OFC [25] consistent with bottom-up and top-down information-processing interactions between these regions during complex ‘higher-order’ decision-making tasks [8], including wilful top-down emotion regulation [8], [18], [21]. The BLA is therefore strongly implicated in mediating competition with the executive PFC over attentional resources.

fMRI is not well-suited to answer the question of baseline attention because it cannot identify what structures are indispensable for a certain function [3]. Lesion studies can sometimes illuminate phenomena fMRI cannot “see”, but subjects with bilateral amygdala lesions are not easy to find. Amygdala lesions that encompass more than just the BLA may have quite different functional effects than selective BLA lesions [26]. A ‘competition for attentional resources’ hypothesis predicts that selective BLA lesions will enhance working memory by alleviating the tonic drain on DLPFC attentional and/or neural resources that automatic bottom-up salience surveillance normally consumes. Here we report our findings of enhanced working memory performance in three UWD subjects with rare selective bilateral BLA calcification but otherwise normal amygdala function.

## Methods

### Ethics Statement

This study was approved by the Health Sciences Faculty Human Research Ethics Committee of the University of Cape Town. All participants provided written informed consent.

### Participants

Three female UWD subjects between the ages of 24 and 35 selected for having no secondary psychiatric or neurological complications were compared with a healthy control group (N = 10) matched for sex, age and education. All subjects live in remote northern South Africa [27]. UWD is an autosomal recessive syndrome traced to a mutation in the extracellular matrix protein 1 gene (EMC1) and our three UWD patients are homozygous for this mutation [27]. All our controls were screened and proved homozygous for the normal variant of the gene. This study was approved by the Health Sciences Faculty Human Research Ethics Committee and all participants provided written informed consent.

### Neuropsychological Assessment

Performance IQ (PIQ), verbal IQ (VIQ) and full-scale IQ (FSIQ) were measured using the Weschler Abbreviated Scale of Intelligence [28]. Based on neuropsychological data collected between 2002 and 2005, Thornton et al. [27] described the entire South African UWD population and demographically matched healthy controls. Neuropsychological and neuroimaging assessments on a sub-sample of these UWD subjects (selected for having no secondary psychiatric or neurological disorders - cf. Thornton et al. [27]) and healthy controls was next performed by us in Cape Town in May 2007. For many of these UWD and control participants coming to Cape Town for MRI scanning and neuropsychological testing was their first journey far from home.

All subjects live in economically impoverished regions where the quality of school education is far below Western norms. It was therefore not surprising to find that this group did not perform well on the Wechsler Adult Intelligence Scale (WAIS-III) [28] which was developed in a First World setting according to Western cultural and educational norms. Both UWD and control IQ test results in May 2007 closely resembled those reported by Thornton et al. [27] i.e. several participants scored in the borderline range. The Wechsler scale purports to measure “the global capacity of a person to act purposefully, to think rationally, and to deal effectively with his environment” [29]. As can be seen in Table 1, most of the participants in our study hold jobs in areas where unemployment exceeds 30% [30]. The problems inherent in using the WAIS-III in a transcultural setting are made starkly apparent by the fact that both Thornton et al. [27] and ourselves in May 2007 (despite excluding subjects with secondary psychiatric or neural pathology) observed many scores in the borderline range.

**Table 1:** Social and occupational status of the participants.

Patient-ID	Social Status
UWD 1	one child, tourism advisor
UWD 2	one child, housewife
UWD 3	own cosmetics sales business
Control-ID	
1	trainee nurse
2	two children, housewife
3	one child, housewife
4	clinic assistant
5	three children, community health worker
6	three children, security guard
7	one child, factory supervisor
8	one child, assistant nurse
9	three children, bank teller
10	one child, security guard

This together with the progressive course of amygdala calcification in UWD made it necessary to test everyone again in 2010. This time, taking note of the WEIRD (Western, Educated, Industrialized, Rich, Democratic) sampling bias issue in human neuroscience [31]–[33] we made several changes in the way the tests were administered.

Participants were now tested:

- In their local environment.
- By a local psychologist who speaks the same Afrikaans dialect as they do.
- Using an abbreviated test, the Wechsler Abbreviated Scale of Intelligence (WASI, which provides for a reliable IQ estimate) [28], because participants reported being overwhelmed by the burden of WAIS-III testing in 2007.
- The WASI verbal tests were translated by local linguists into the local Afrikaans dialect.

The 2010 IQ scores (reported in Tab. 2 below**)** show a global increase of approximately 10% with everyone now falling into the low-normal range. The fact that the changes we made brought about this improvement are in line with the WEIRD discussion [31]–[33]. Specifically, we attribute this improvement to the fact that in 2007 participants were tested in a strange environment and by an unfamiliar person of a different race (especially problematic in post-Apartheid SA), culture, dialect and socioeconomic position. It can however be stated with confidence that the 2010 IQ scores are still an underestimate of the participants’ capabilities. Firstly, although the difference in conditions between 2007 and 2010 made a significant difference, we were obviously unable to overcome all cultural, language and educational biases inherent in the WASI [34]. Secondly, even these improved scores are inconsistent with the participants’ ability to compete very favorably for semi-skilled jobs under extremely adverse economic conditions.

### Neuroimaging

Structural and functional MRI scans were acquired with a Siemens Magnetom Allegra 3-Tesla head-only scanner at the Cape Universities Brain Imaging Centre (CUBIC) in Cape Town, South Africa.

### Structural MRI Assessment

Structural whole brain T2-weighted MRI scans were obtained with 1 mm isotropic resolution, TR = 3500 msec, and TE = 354 msec.

#### MRI analysis

Based on MR-images the precise borders between amygdalae and neighboring structures, or between the subnuclei of the amygdala, cannot be established [35], [36]. Therefore, we normalized the T2-weighted scans of all 3 UWD subjects to the template of the Montreal Neurological Institute (MNI) using the unified model as implemented in SPM5 [37]. This unified model combines tissue classification, bias correction and nonlinear transformations into one parallel procedure, which optimizes normalization of lesioned brains [38]. Subsequently the extent of the calcifications was determined with the 3D volume-of-interest feature implemented in MRIcroN [39]. The resulting volumes and the lesion-overlap (voxels that were represented in all individual lesion volumes) were mapped onto cytoarchitectonic probability maps of the basolateral-, central-medial- and superficial amygdalae [35].

In this method, that is implemented in the SPM5 anatomy toolbox [40], a volume of interest (VOI) is superimposed onto a cytoarchitectonic probability map of the amygdala and hippocampus [35]. This map is based on microscopic analyses of ten postmortem human brains and follows a generally accepted division of the human amygdala in three sub-regions. The first is the central-medial amygdala (CMA), which consists of the central and medial nuclei. The second is the basolateral amygdala (BLA), which includes the lateral, basolateral, basomedial, and paralaminar nuclei, and the third is the superficial (or corticoid) amygdala (SFA), which includes the anterior amygdaloid area, amygdalopyrifom transition area, amygdaloid-hippocampal area, and the cortical nucleus [35]. This method assigns to any given voxel a value representing the probability that it belongs to an underlying structure. These are derived from an overlap analysis of ten postmortem brains, and are therefore divided in ten separate probability classes ranging from 10% to 100% probability. For each probability-class of each structure that shares voxels with the VOI, the ‘observed versus expected’ class representation is computed. This value represents how much more (or less) that class is observed in the VOI compared to what could be expected from the entire probability map of that structure, and is computed with the following equation:
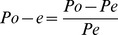
Whereby *P_o−e_* represents the ‘observed versus expected’ class representation, *P_o_* represents the percentage of VOI voxels in that class, and *P_e_* represents the percentage of voxels from that class in the whole cytoarchitectonic map of that structure. The outcome values thus indicate which class is over-represented in the VOI relative to the whole cytoarchitectonic map.

To estimate how well the lesion volumes fit the underlying structure, *P_excess_* values are computed using the following equation:
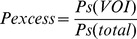
Whereby *P_s(VOI)_* represents the average cytoarchitectonic probability of the voxels that are shared by the structure and the VOI, and *P_s(total)_* represents the average probability of the whole structure’s cytoarchitectonic map. These values thus represent how much the average probability of the overlapping voxels exceed the overall probability distribution of that structure, and thus indicate whether the VOI overlaps with relatively high or low probability classes of that structure. In other words, *P_excess_* represents how ‘central’ the location of the VOI is relative to that structure’s cytoarchitectonic map, whereby *P_excess_* >1 indicates a more central, and *P_excess_* <1 a more peripheral location [41].

### Functional MRI Assessment

Functional whole brain MRI scans were obtained with a 2D-EPI sequence with 36 slices in interleaved-ascending order, 3.5 mm isotropic resolution, Flip-angle = 70°, TR = 2000 msec, TE = 27 msec, and EPI-factor = 64. The first 4 volumes were acquired prior to the start of the emotion-matching task, and discarded from the analyses.

**Figure 1:**
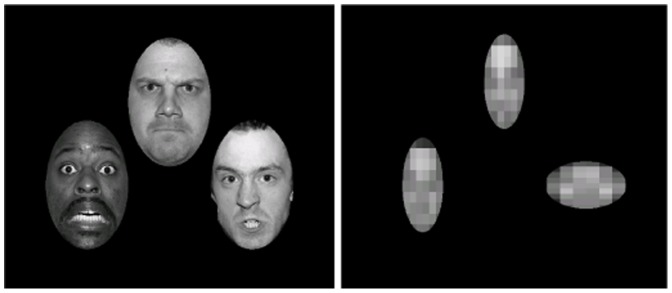
Screenshots of the emotion and shape matching task stimuli. Each screen remained visible for 5 seconds during which time subjects responded by pressing the button in their left or right hand to record which lower image (left or right) matches the upper image.

To assess amygdala functionality, we employed a well-validated emotion-matching task adapted from Hariri and colleagues [42]. The original version of this task has reliably assessed individual differences in amygdala reactivity [43], and has successfully differentiated between dorsal and ventral amygdala activity [44]. In this task participants match facial emotional expressions, or abstract oval shapes, by choosing one of two pictures in the lower part of a display (either an angry and a fearful face, or a horizontal and vertical oval shape) to be similar (emotion or shape) to a picture on top of the same display (Figure 1). To increase the cultural validity of this task for our participants, face stimuli were adapted from the NimStim set of facial expressions and included Caucasian as well as African actors [45]. Six actors (three female) were selected based on the emotional validity ratings included with the NimStim-set. Gray-scaled, oval cut-outs including the whole face were used as stimuli and the shape-stimuli were constructed by scrambling the facial stimuli to match visual contrast levels between emotion and shape matching trials.

The task was presented in a blocked design, with 5 shape-matching blocks interleaved with 4 emotion-matching blocks. Each block consisted of 6 trials of 5 secs each, resulting in a block-length of 30 secs. Emotion matching trials always included faces of one gender only, and all faces were presented equally often as target, match or non-match in fully randomized order. Each block was preceded by the instruction ‘match emotion’ or ‘match shape’ (in Afrikaans translation) for 2 sec, making a total task duration of 288 sec. Stimulus displays were back-projected on a screen within the scanner-room and visible to the participant through a mirror. Participants practiced with the procedure before the actual task was started and care was taken that they fully understood the procedure. Participants responded to the task trials by pushing a button with either the left or right hand, corresponding to the position of the match-stimulus, which was balanced for both emotion and shape.

#### FMRI analysis

Functional MRI data analyses were performed with SPM5 [37]. For each participant all volumes were realigned to the first volume using a least-squares rigid-body 6-parameter transformation, and coregistered to the structural T2-weighted volume based on maximization of mutual information [46]. Subsequently, the resulting functional images were normalized to MNI-space using the parameters obtained from the structural analysis as described in the *Structural assessment* section, and smoothed with a FWHM Gaussian kernel of 8×8×8 mm.

A general linear model [47] was applied to the resulting images to investigate the effects of emotion versus shape matching. Contrast-maps for both conditions were obtained using a 30 sec boxcar function convolved with a hemodynamic response function as implemented in SPM5. To reduce unexplained variance, the realignment parameters and a discrete cosine transform high-pass filter (cut-off 128 sec) were entered as regressors of no interest. Second-level analysis was performed by contrasting both conditions with a paired-samples T-test resulting in a T-map of the emotion-minus-shape contrast.

Functional activation of the amygdala was assessed unilaterally in each hemisphere within two regions of interest (ROI's): the basolateral (BLA) and the combined central-medial (CMA) and superficial (SFA) amygdalae. ROI's were constructed based on the cytoarchitectonic probability maps as implemented in the anatomy toolbox for SPM5 [35], [48]. We applied an extent-threshold of 10 voxels, and significance level was set at p<.05 (false-discovery-rate (FDR) corrected). This rather lenient threshold is justifiable given that we presently use these data to assess whether the amygdala's subregions are functional in general, and not what their function would be on this task employed as an emotion-discrimination task.

### Working Memory and Anxiety

Outside of the scanner, the WAIS III [28] digit span forward task (DSF) was administered to all subjects in their mother tongue of Afrikaans. In the DSF task a sequence of digits is read aloud to the participant who must then verbally repeat the sequence. The first item comprises a sequence of only two digits, the second item three digits, the third item four digits, and so on. There are two trials per item (e.g. item 3 comprises two separate four digit sequence trials). Subjects score one point for each correct trial. The task continues until the subject fails to correctly repeat both trials of any item. A score of 12 for example, requires perfect repetition up to the end of item six (two correct seven-digit spans), or one correct trial on item six (one correct seven-digit span) plus one correct trial on item seven (one correct eight-digit span) followed by no correct trials on item eight (no correct nine-digit spans). Records from 2003, the only other occasion the DSF task was administered to these UWD subjects and matched controls, were also retrospectively examined (Unpublished observations from the study reported in Thornton et al. [27]). Note that although the WAIS III was administered to the patients and controls in 2007 as described above, the complete battery was not administered on that occasion and the DSF task was one of the tests omitted. Thus the UWD group did the DSF task on only two occasions (2003 and 2010). On each occasion the control groups were not the same individuals so the controls only performed the task once. Since there is no reason to suspect that either group performed the DSF task in any other context, we do not believe a training or practising effect is an issue of concern.

In order to control for possible emotional trait differences between UWDs and controls the Spielberger State-Trait Anxiety Inventory (STAI-T) was administered. To control for possible emotional differences during the digit span forward task, subjects were asked to rate their subjective feelings of stress and tension on a scale from one to one hundred after performing the working memory task. As was the case for the digit span forward task, all these control measures were administered outside of the scanner.

### Statistical Methods

Non-parametric Mann-Whitney U tests were performed on all behavioural data. Exact two-tailed p-values are reported.

## Results

Performance IQ, verbal IQ and full-scale IQ scores for all subjects fell within the low-normal range (Table 2) and no significant group differences were found: PIQ (Z = −.852, p = .469), VIQ (Z = −.682, p = −.573), FSIQ (Z = −.426, p = .692).

**Table 2:** Age, schooling, and Wechsler Abbreviated Scale of Intelligence for each UWD subject and controls. PIQ, performance IQ; VIQ, verbal IQ; FSIQ, full-scale IQ.

		UWD group				Control group	
		UWD 1	UWD 2	UWD 3	Mean	Mean	S.D.
Age		24	31	35	30.0	31.8	6.8
VIQ		95	84	93	90.7	88.7	3.6
PIQ		98	86	85	89.7	90.3	3.9
FSIQ		97	84	87	89.3	88.0	2.5
Years Schooling		12	9	12	11.0	10.6	1.3

**Figure 2:**
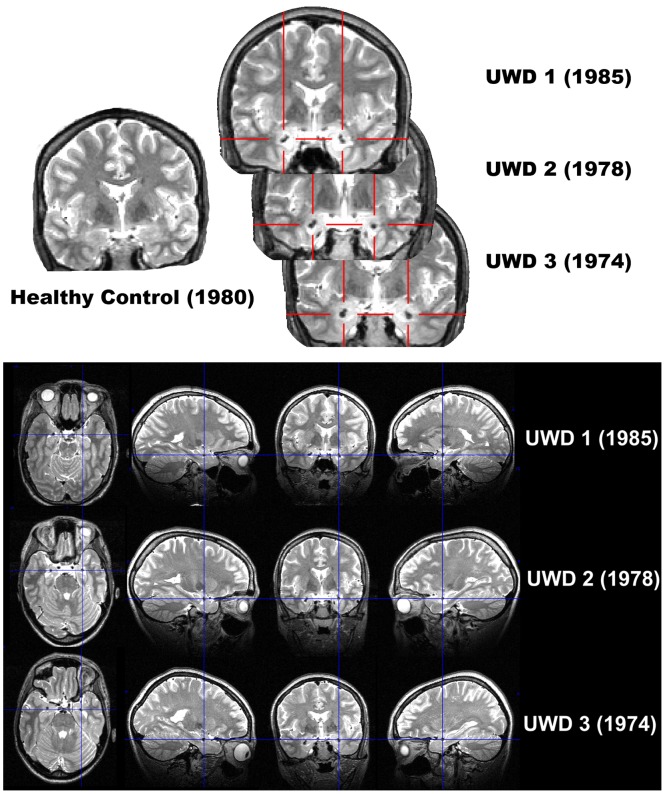
Structural MRI scans showing bilateral amygdala calcification in UWD. **Figure 2a:** Coronal view T2-weighted MR-images of the three UWD subjects and one control subject with year of birth. Crosshairs indicate calcified brain damage. **Figure 2b:** T2-weighted MR-images in all three planes of the three UWD subjects. Crosshairs indicate the location of calcified brain damage bilaterally.

**Figure 3:**
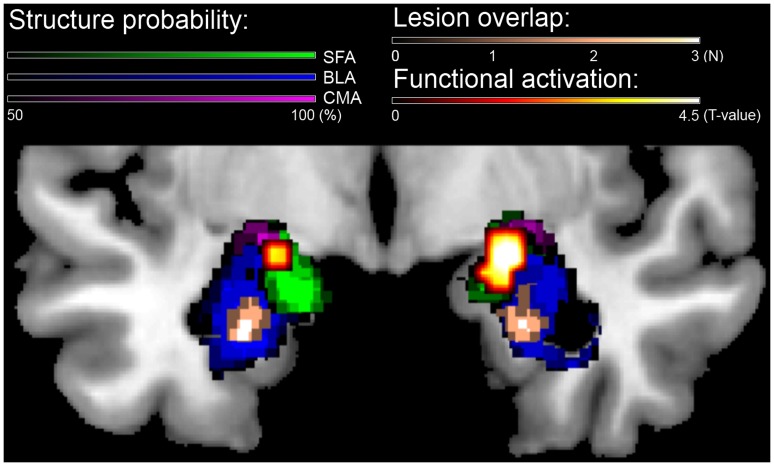
Structural and functional assessment of the bilateral amygdala in our group of three Urbach-Wiethe Disease subjects. Plotted are the cytoarchitectonic probability-maps of the amygdalae thresholded at 50%, structural lesion overlap, and functional activation during the emotion-matching task (contrast: Emotion>Shape matching, significant clusters p<.05, FDR-corrected for paired samples t-tests within ROI’s analysed separately for each hemisphere) on a template brain. The structural method indicates that the lesions of the three UWD subjects are located in the basolateral amygdala (BLA), while the functional method shows activation during emotion matching in the superficial amygdala (SFA) and central-medial amygdala (CMA), but not in the BLA.

**Figure 4:**
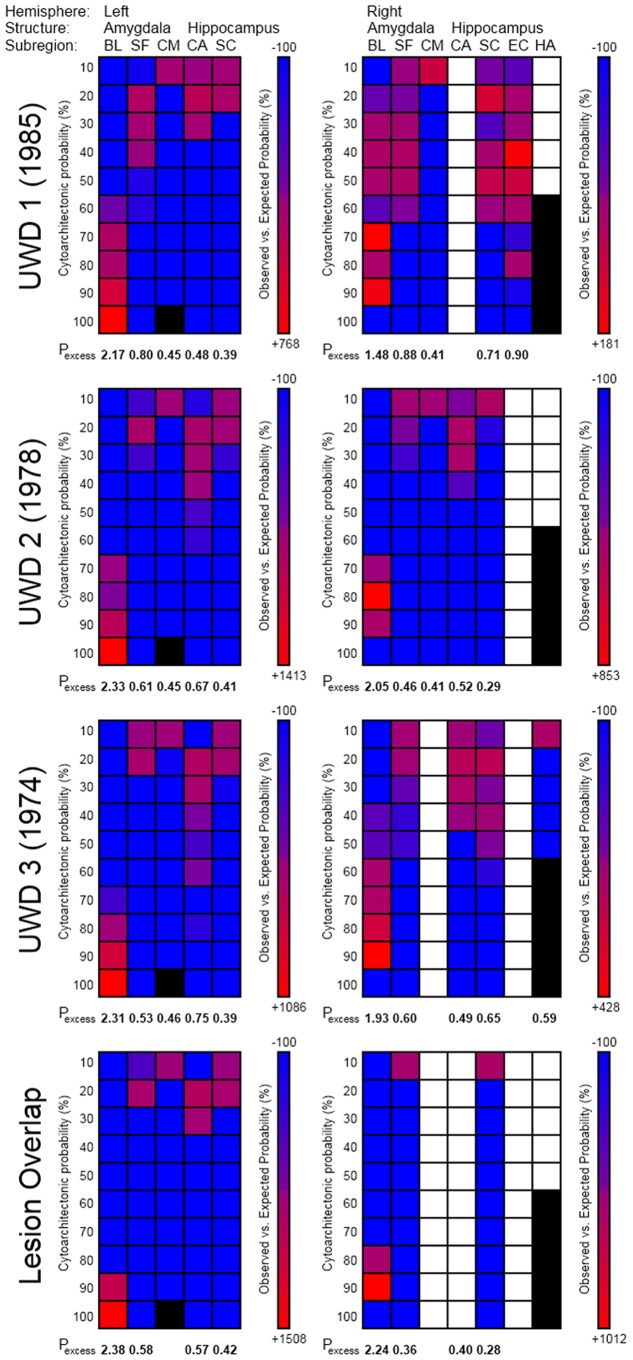
Observed versus expected probability matrices for the individual brain lesions and their overlap. Columns are the observed brain areas, and rows their cytoarchitectonic probability classes. Colors indicate the relative over- (red) or under- (blue) representation of a structure-class in the lesion volume. White indicates no overlap between lesion and structure probability map, and black indicates probability classes that are not represented in the cytoarchitectonic map. P_excess_ values indicate how much more likely a structure was observed in the lesion volume as could be expected from its own probability distribution, and thus reflect how central to the area the lesion volume is. BL = Basolateral, SF = Superficial, and CM = Central-Medial, which are all amygdala subregions, and CA = Cornu Ammonis, SC = Subicular Complex, EC = Entorhinal Cortex, and HA = Hippocampal-Amygdaloid Transition Area, which are all subregions of the hippocampus.

T2-weighted MRI scans and structural assessment of the bilateral amygdalae are shown in Figures 2, 3 and 4. Figure 4 shows that P_excess_ reached values of, in order of age, 2.17, 2.33, and 2.31, for the left-sided, and 1.48, 2.05, and 1.93, for the right-sided BLA. P_excess_ values for the CMA were all <.5. For the lesion-overlap volumes P_excess_ reached values of 2.38 and 2.24 for the left and right BLA respectively, while P_excess_ values for all other structures was <.6. We conclude from these data that all three UWD subjects have calcified brain-tissue in the BLA, while the CMA seems unaffected.

As anticipated, control subjects showed robust functional activation of all three amygdala sub-regions in response to the emotion versus shape matching task. For the UWDs, FDR-corrected (p<05) functional activation on the emotion versus shape matching contrast is shown in Figure 3. This ROI-analysis revealed no significant clusters in the bilateral BLA, but in the ROI constructed from the other subregions of the amygdala (i.e. the ROI of the combined CMA and SFA analysed separately for each hemisphere) significant clusters of 26 and 100 voxels (left and right hemisphere respectively, p = .035) were found. Thus, no activation was observed in the BLA, but CMA and SFA still seem to be functional.

For UWD1, UWD2 and UWD3 emotion-matching accuracy was 96%, 79% and 96% and shape-matching accuracy was 80%, 97% and 77% during the fMRI task respectively. Individual binomial tests with test proportion = 0.5 (chance-level) established that all three UWD subjects performed the matching task properly (all p’s <0.007).

**Figure 5:**
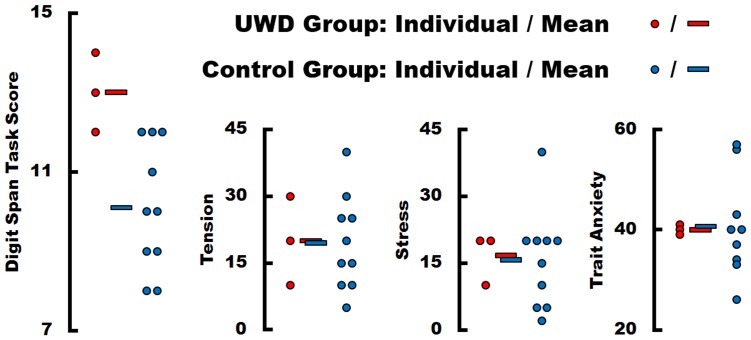
Working Memory scores (for 2010) on the Digit Span task showing superior performance of three UWD subjects with bilateral basolateral amygdala calcification compared to ten normal controls. Subjects were asked to rate on a scale of 1 to 100 how much Stress and Tension they felt during the task. Trait anxiety results for the Spielberger Trait Anxiety Inventory are also shown.

Working memory performance measured on the DSF task was significantly better in UWD subjects than controls (Z = −2.234, p = .014) (Figure 5). To get a feel for these differences, only two control subjects scored 100% on item 6 (two correct seven-digit spans) whereas all three UWD subjects scored 100% on item 6. One control subject and one UWD patient (UWD3) scored 50% on item 7 (one correct eight-digit span) and one UWD patient (UWD1) scored 100% on item 7 (two correct eight-digit spans). No subjects scored above zero for item 8 (nine-digit span). DSF results from 2003 show a very similar pattern (Table 3). The two older UWD subjects outperform all their controls, while the youngest UWD subject who was seventeen years old at the time and still in school, scores similar to her controls.

**Table 3:** Digit Span results for the three UWD subjects and controls from 2003 (Unpublished observations from the study reported in Thornton et al. 2008 [27]).

	DSF score	range	max span
**UWD 2**	10		7
**UWD 3**	11		7
**Controls (n = 5)**	8.4	8–9	6
**UWD 1**	8		5
**Controls (n = 4)**	8.25	7–9	6

Above, Digit Span results from 2003 for the two older UWD subjects and mean score of matched controls. Below, Digit Span results for the younger UWD subject and mean score of matched controls.

Measures of trait and state anxiety showed no significant group differences: STAI-T (Z = −.279, p = .864), Stress (Z = −.171, p = .937), Tension (Z = −.446, p = .692) (Figure 5).

## Discussion

UWD is an extremely rare disorder [27]. Worldwide, neuropsychological data for less than 50 cases has been published, the majority stemming from the South African population our three subjects derive from [27], [49]. To date there are only four cases for which both WM performance and structural MRI brain lesion data exist. The three cases reported here, the only cases for whom amygdala functional MRI data has ever been described, almost doubles this figure.

Although enhanced WM performance has not previously been reported in UWD, our cases differ in several respects from previous studies reporting normal WM in UWD [50]–[54]. Firstly, in our sample, all amygdala lesions are focal and confined entirely within the boundaries of the BLA bilaterally (Figures 2, 3 and 4). All four other UWD cases for whom data is available have either more extensive BLA damage [53], [55], or damage which occupies the whole amygdala bilaterally [54], or damage which occupies the whole amygdala and encroaches upon adjacent structures bilaterally [13], [53]. Secondly, none of our subjects have secondary psychopathology and although the same is true for the other four cases, two of them do evidence grossly abnormal social/affective behaviour such as extreme invasion of personal space and profound hypophobia [56]–[58]. Our UWD subjects show none of these or any other noticeable social/affective abnormalities. Thirdly, while SFA and CMA function is spared in our subjects, no evidence of functional activation within these sub-regions has been demonstrated for any other UWD subject so far. Lastly, two of the other four UWD subjects are diagnosed with epilepsy secondary to cerebral calcification, one being on chronic medication for grand mal epilepsy [55] while both experience frequent epileptic auras [54]. Our UWD subjects have no history of epilepsy or any other brain disease and are not on chronic treatment for any medical condition. One or more of these differences could account for the fact that facilitation of working memory has not previously been reported in UWD.

Prior results indicate that our findings are not attributable to chance. As shown in Table 3, DSF data for these three UWD subjects from 2003 largely replicates the current findings (unpublished data from the study reported by Thornton et al. [27]). This was the only previous occasion on which the DSF task was administered to these UWD subjects and matched controls. Although UWD1, who was only 17 years old at the time, did not perform better than controls in 2003, she outperforms all other participants in the current study. That she only managed to accurately recall a maximum span of five digits in 2003 (compared to 8 digits twice in 2010) suggests that she did not perform to the best of her ability on that occasion. This would be in keeping with the fact that the same factors responsible for the overall improvement in IQ scores between 2007 and 2010 (see Neuropsychology under Methods above) also apply to 2003. 2003 was also these subjects’ first experience of neuropsychological testing. It should also be noted that our UWD subjects and controls are particularly well matched for age, intelligence, and socioeconomic status as well as for physical and social environment. Lastly, none of the 2003 control subjects participated in 2010 which further validates the replicated findings in the two older UWD subjects. All of this, together with the fact that the UWD subjects’ DSF performance is quite impressive by any standards, effectively excludes the possibility that the effects derive from chance or low-performing control groups.

The significance of our findings lies in the fact that brain damage almost always causes functional impairment. Enhanced WM performance is therefore somewhat paradoxical. One possible explanation is that the working memory task induced less performance anxiety in UWD subjects than in controls, hence causing less emotional interference in cognitive processes subserving working memory. This possibility is consistent with a recent working memory study in which normal subjects reported negative feelings in response to increased cognitive load and the intensity of negative emotion correlated positively with amygdala activation and negatively with working memory performance [15]. Evidence of impaired episodic memory modulation by emotion in UWD subjects [55], [59] is also consistent with a ‘decreased emotional interference’ hypothesis. However, as reported above, we controlled for possible differential emotional reactivity between UWD subjects and controls. Measures of task-induced stress and tension as well as trait anxiety (STAI-T) revealed no significant state or trait differences between the UWD group and controls (Figure 5). This absence of anxiety differences between UWD subjects and controls is in keeping with previous reports that amygdala damage does not affect subjective arousal or valence ratings of emotional stimuli [55], [59], [60]. The ‘decreased emotional interference’ hypothesis does therefore not explain the enhanced working memory findings.

It should also be noted that the paradoxical functional facilitation of WM observed in these three UWD subjects with bilateral BLA damage occurred in the absence of any salient stimuli. The effect of salience on their working memory performance is therefore unknown. Future research in these subjects should address this question.

A general term for enhanced performance following brain damage is *paradoxical functional facilitation* [61]. Paradoxical functional facilitation calls for a more subtle understanding of brain function than traditional localisation models reminiscent of phrenology. The emerging model [4], [62], [63] is premised on the fact that neurons communicate in a language of only two words: excitation and inhibition. At the level of the whole brain we must also think in terms of “inhibitory and excitatory interactions between a number of diverse neural circuits rather than the operation of discrete neural systems in isolation” [61].

An interactive model makes it easy to imagine how paradoxical functional facilitation effects might arise, how damage to region A might result in enhanced performance of a function subserved by region B. If prior to any damage region A is directly or indirectly interfering with region B, subsequent damage to region A might stop this interference. Reduced interference has previously been proposed as an explanation for observations of paradoxically enhanced working memory in amnesic patients. To elicit this effect, subjects are first ‘primed’ with information designed to interfere with further information subsequently presented as part of a working memory test. Amnesic patients are less able than controls to remember the primed information, hence less interference and better performance in the working memory test [61].

The current study differs from studies showing paradoxical functional facilitation of working memory in amnesic patients in that the subjects have no amnesia, the working memory task requires no priming and the identical focal BLA lesion is present bilaterally in all UWD subjects. Together these factors directly implicate the BLA in mediating an internal source of interference on working memory. What could such internally generated interference be?

The evolution of enhanced working memory allowed working attention to be directed away from the broader environment to be focused on a complex task or to be projected into the future or past [64]. Although this is perilous in a natural setting replete with danger, the PFC is able to integrate much more information than the BLA and is sometimes better positioned to assess threat. It is therefore advantageous for PFC to be able to override BLA “false alarms” during goal-oriented cognition. Nevertheless, since orientation to salience is rapid, involuntary and effortless, whereas executive inhibition of bottom-up interference is effortful, it is clear that the BLA retains the ability to override executive functions and bring attention back to the salient present.

This fundamental functional difference between the BLA salience hub and executive attention is mirrored by neuroanatomy in that DLPFC and the amygdala as a whole (including the BLA) share few direct connections [25], [65]. Neuroimaging evidence suggests that functional connectivity between these regions during working attention is via orbitofrontal cortex (OFC) [15] and structural studies show that among all PFC and amygdala subdivisions, OFC and BLA are most massively and reciprocally connected [25]. There is also strong resting-state functional connectivity between laterobasal amygdala and OFC [24], [62]. Although BLA-OFC communication is integral in bottom-up salience signalling and top-down emotion regulation [8], [21], [66] the absence of direct connectivity between executive DLPFC and amygdala underlines the idea that DLPFC is never in total command of the brain’s attentional resources.

The ascending BLA-OFC pathway is considered to be crucial in updating OFC of changes in salience [8], [66]. OFC however, does not merely relay salience signals from BLA to DLPFC. Being better-informed than the amygdala to evaluate threat [8], the OFC can itself immediately commandeer cognitive control and override involuntary amygdala-mediated defence reflexes in order to orchestrate more sophisticated responses based on explicit knowledge [8], [66]. As Ghashghaei and Barbas have written, the basolateral amygdala and the orbitofrontal cortex together “appear to have a global overview of the environment, which likely is necessary for processing and remembering the emotional significance of stimuli” [25].

This switch in the locus of working attention from executive attention mediated by dorsal PFC to the salience-sensitive BLA-OFC circuit again emphasizes competition between working attention and bottom-up salience-sensitive networks. Thus, although human intelligence may have hinged upon the evolution of improved working memory enabling efficient wilful DLPFC inhibition of salience “noise” emanating from the amygdala, the high degree of structural insulation and functional competition between DLPFC on the one hand and the bi-directional BLA-OFC circuit on the other supports the idea that ceaseless competition for attentional resources between working memory and salience surveillance remains a fundamental survival feature of the human brain.

In conclusion, we show paradoxical functional facilitation of working memory in three UWD subjects with selective bilateral BLA lesions. This suggests that ongoing salience surveillance by the BLA exacts a tonic cost on executive attentional resources at the expense of working memory. The present study is to our knowledge the clearest evidence to date that the BLA is an essential node mediating competition between salience and executive networks for attentional resources in the brain.
